# Phytochemical and Pharmacological Review of Da Chuanxiong Formula: A Famous Herb Pair Composed of *Chuanxiong Rhizoma* and *Gastrodiae Rhizoma* for Headache

**DOI:** 10.1155/2013/425369

**Published:** 2013-08-27

**Authors:** Lu Wang, Jinming Zhang, Yanlong Hong, Yi Feng, Meiwan Chen, Yitao Wang

**Affiliations:** ^1^State Key Laboratory of Quality Research in Chinese Medicine, Institute of Chinese Medical Sciences, University of Macau, Macau; ^2^Engineering Research Center of Modern Preparation Technology of TCM of Ministry of Education, Shanghai University of Traditional Chinese Medicine, Shanghai 201203, China

## Abstract

Chronic headache such as migraine and nervous headache has become one of the most common locations of pain and one of the most difficult diseases to recover due to its numerous causes and inconvenience to keep acesodyne administration for a long time. However, there are a series of treatment theories and herbal formulas for this disease in traditional Chinese medicine (TCM), in which Da Chuanxiong formula (DCXF), a herb pair composed of *Chuanxiong Rhizoma* (CR), Chuanxiong in Chinese, and *Gastrodiae Rhizoma* (GR) called as Tianma in China, is a greatly classic representative. This formula has been used for headaches via dispelling wind pathogen and dissipating blood stasis for many years in TCM. In recent years, the efficiency and representativeness of DCXF have garnered many researchers' attention. To reveal the compatibility mechanism and develop innovative Chinese herb, herein ethnopharmacological relevance, chemical characters, and pharmacological actions of DCXF are detailed. It is expected to give a comprehensive interpretation of DCXF, namely, Chuanxiong Tianma herb pair (CTHP), to inherit the essence of herb pair and innovate drug delivery system of this prescription.

## 1. Introduction

With the long-term survival fight against severe environment, early humans in China acquired the knowledge to treat disease even death by applying natural material [1, 2]. In traditional Chinese medicine Classics, such as famous *Sheng Nong's Herbal Classic* in Han Dynasty and *Compendium of Materia Medica* in Ming Dynasty, there are amounts of records about disease treatment experience using natural plants, animals and minerals, which were completely called as traditional Chinese medicine (TCM) [3]. Through many years of vigilant observations, experience, and trial and error experiments, TCM has formed the basis of sophisticated traditional medical systems as the health security for Chinese people since the dawn of time. Additionally, with the growing research attention from worldwide, TCM has developed an increasingly important medical system for not only China but also rest of the world and continued to provide humankind with dawn of new remedies for modern critical disease [4–6]. 

In the early stage medical practices, single herb was used as predominant remedy for diseases. With the accumulation of further therapeutic experience, TCM practitioners gradually realized the complexity of pathogenesis and found some interesting compatibility art among herbs, developing its cosmologic principle of Chinese philosophy including holism, differentiation, yin/yang, and the five elements. Until now, multiherb therapy as an essential component of traditional medicine systems still has been utilized. Herb pair, the unique combinations of two relatively fixed herbs in clinic, is the most fundamental and the simplest form of multiherb therapy to achieve a specific efficacy by a unique methodology [7]. Many herb pairs were recorded in the *Treatise on Febrile and Miscellaneous Diseases* and in the *Synopsis of the Golden Chamber*. Though there are several methods of herbs compatibility with different aims, which are called as “seven relations of Chinese Medicine”, such as singular application, mutual promotion, mutual assistance, mutual restraint, mutual detoxication, mutual inhibition, and mutual intoxication [8], herb pairs for mutual potentiation occupy mostly according to Chinese records and the classic books about herbs in China. Mutual potentiation, also called as mutual promotion, showed significantly better pharmacological efficacy than when the herbs were used individually, being applied in many famous herb pairs such as Danggui Buxue Decoction (Astragali Radix and Angelicae sinensis Radix) [9, 10] and Dan Qi Fang (Salviae miltiorrhizae and Panax notoginseng) [11]. 

Da Chuanxiong Formula (DCXF) is not only a famous Chinese medicinal prescription but also a classic herb pair for mutual potentiation composed of two herbs, namely, Chuanxiong (*Ligusticum chuanxiong* Hort) and Tianma (*Gastrodiae elata* Bl.) with a crude weight ratio of 4 : 1, used for headache caused by blood stasis and wind-heat syndrome. This herb pair, first recorded in “*Xuan Ming Lun Fang*” in Jin Dynasty, has been widely used to promote blood circulation, balance the liver and extinguish wind, eliminate pathogens, and dredge the collaterals to stop pain with thousands of years' clinical experience. In modern medicine, its antimigraine effect has been definitely confirmed [12–15]. Now, various dosage forms of this herb pair on the market such as capsules, pellets, oral decoctions, and granules, are mainly indicated for a blood stasis type of headache. However, the synergism mechanisms of the formulae have not been elucidated, and research progress of this combination has rarely been summarized.

Herein, the research status of Chuangxiong-Tianma herb pair (CTHP) namely, DCXF is discussed in terms of its ethnopharmacological relevance, material basis, and pharmacological actions by means of modern analysis methods. It is expected to give a comprehensive interpretation of DXCF in order to inherit the essence of herb pair and innovative drug delivery system of this prescription.

## 2. Ethnopharmacological Relevance

### 2.1. Traditional Background of Herbs

*Chuanxiong Rhizoma* (CR), Chuan xiong in Chinese, is the dried rhizome of *Ligusticum chuanxiong* Hort., which belongs to the Umbelliferae family and its optimal harvest time is in the period from April to May which has been indicated as the best time for the accumulation of active constituents, such as volatile oils, ferulic acid, and alkaloids [25–27]. The resources of both wide and cultivated CR are wide distributed in Pengzhou, Xindu, Chongzhou, and Dujiangyan in Sichuan province of China [28, 29]. Zhu et al. compared the contents of tetramethylpyrazine and ferulic acid, which are two main active constituents of CR, in different CR samples from different seasons as well as different producing areas using HPLC, finding that the contents of teramethylpyraine and ferulic acid were significantly different [27]. It has been used for thousands of years in traditional Chinese, Japanese, and Korean folk medicine [30]. According to traditional Chinese medicinal theory, CR could promote blood and Qi circulation and dispel wind and relieve pain in TCM [31]. For its reputation of facilitating blood circulation and dispersing blood stasis [32], CR is not only used for medicinal purpose, like treating atherosclerosis [33], ischemic stroke [34], vasodilation [35], and thrombus formation [36], but also for food preparation and health care products, such as mutton soup [37], tobacco flavor additive, and natural preservative [38].

*Gastrodiae Rhizoma* (GR), Tianma in Chinese, is the dried rhizome of *Gastrodiae Elata* BI, which belongs to the Orchidaceae family and is widely used as a traditional medicine in China, Japan, and Korea. Known as a kind of commonly used and expensive traditional Chinese medicine, GR is mainly produced in Sichuan, Yunnan, Guizhou, and Shanxi province in China [39, 40]. In humid environment, GR could make a good breeding and it commonly harvested in early winter or spring, for it lies dormant during these specific times to maintain its active substances [40, 41]. By using HPLC-DAD and HPLC technique, the chromatographic fingerprint of GR was developed to compare the effective contents of GR from different habitats and founds have good similarity of fingerprints with 14 different producing areas of China, except for the GR in Lueyang, Shaanxi province [42]. In Chinese medicine, GR is one of the most important herbs used for balancing the liver and extinguishing wind for the efficacies of moistening viscera, calming and protecting human body, calming liver and suppressing liver-yang, and calming the wind and activating collaterals [20]. In *Sheng Nong's herbal classic*, a famous TCM classic, GR is considered as one of the top grade drugs, for it has rejuvenating effects and nontoxic property, which can be used without harm for a long time. For thousands of years, in China, GR has been used to treat diseases such as headache, dizziness, paralysis, and convulsion and enhance health by using alone or combining with other Chinese herbs [18].

### 2.2. Clinical Use of DXCF

In TCM, it is generally believed that human heads inclined to be attacked by pathogenic wind, leading to headache. In Song Dynast, traditional Chinese medicinal masters thought that expedite blood circulation could dispel pathogenic wind in body. Thus, it has been one of the most important principles to treat headache in TCM, such as acupuncture, moxibustion, and herbs. Due to the dramatic effect on Qi promotion in blood of CR and the significant effect on internal wind elimination of GR, like the previous description, DCXF, has the undoubted therapeutic effect for headache caused by blood stasis and wind-heat syndrome [22]. Nowadays, DCXF has been used in treating different types of headaches, such as migraine, angioneurotic headache, and the headache caused by endogenous of liver fire and stagnation of the liver-Qi [16, 17, 19, 21, 23, 24, 43, 44]. Interestingly, DCXF could effectively control the headache and relieve the patient's suffering without obvious side effects. Based on pharmacological investigation, DCXF could effectively enhance the cerebrovascular elasticity, change cerebral blood supply, and adjust the instability of cerebrovascular promotion, which had advanced functions for improving clinical symptoms [45]. 

In addition, DCXF also has many other positive curative effects for curing cervical spondylosis [46], cerebral arteriosclerosis [47, 48], ischemic vertigo symptoms [49], and hypertension [50].

## 3. Chemical Properties

All these bioactivities are attributed to the chemical constituents of DCXF. Thus, to understand the material bases of herb pairs is necessary for annotating the compatibility theory and developing it in TCM. In order to get the comprehensive phytochemical information, the chemical constituents of CR and GR will be introduced briefly at first, followed by compatibility behavior of DCXF. 

### 3.1. Chemical Constituents in *Chuanxiong Rhizoma*

*Chuanxiong Rhizoma* (CR) was proven to have many active ingredients, such as phthalide compounds, phenolic acid, essential oil, and alkaloid (Table 1). To date, it has been analyzed that nine volatile oil compounds, 12 kinds of alkaloids, 16 kinds of phenolic acid derivatives, and 33 kinds of lactone components are isolated from CR, which contains four main effective components: ligustilide, tetramethylpyrazine, ferulic acid, and senkyunolide A [30, 51]. Meanwhile, polysaccharide, ceramides, and a variety of other small molecule compounds of lipids are also found as the active compounds in CR.

### 3.2. Chemical Constituents in *Gastrodiae Rhizoma*

*Gastrodiae Rhizoma* (GR) has some main active ingredients: phenols, polysaccharides, microelement, organic acids, and so forth (Table 2). It is known that GR has been intensively isolated and identified to have 20 phenolic compounds and related glycosides, 9 organic acids and related esters, 3 sterols and sterolines, and multiple small organic molecular compounds of furan aldehydes and fatty acids. Evidenced by the recent to relevant reports, gastrodin is considered to be the most effective compound in GR [52–55].

### 3.3. Chemical Constituents in the DCXF

**Figure 11:**
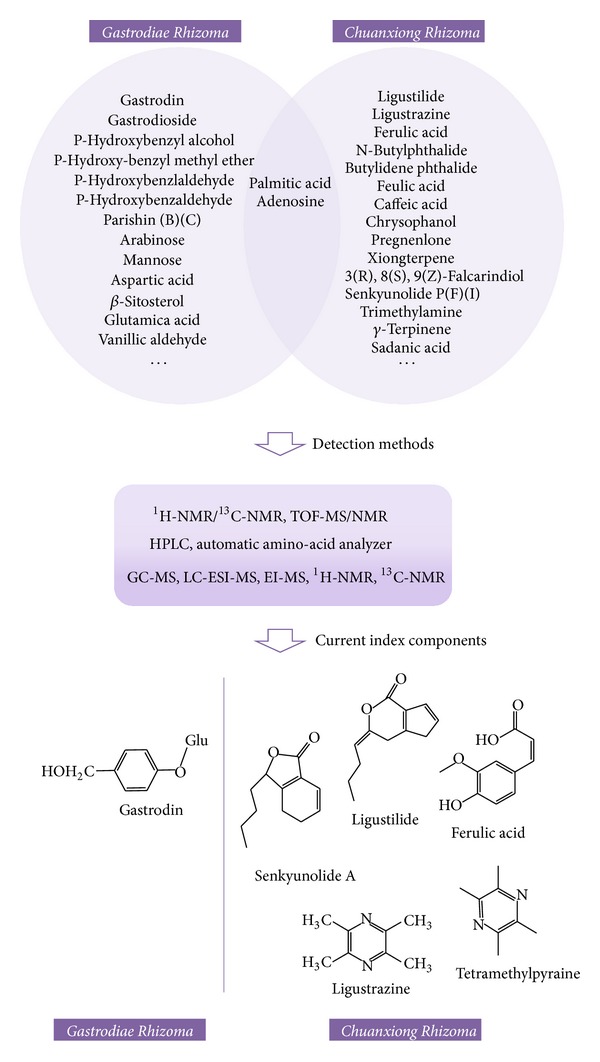
Chemical structures of current index components in Dachuanxiong Decoction.

As earlier discussed, synergistic effects of herb pair would not be produced by simple combination of compounds from two herbs. Based on constituents in single herb, currently, several studies on DCXF have been investigated on its bioactive materials. Researchers have confirmed that ferulic acid, ligustilide, gastrodin, and other components are the active components of treating migraine in Dachuanxiong Decoction (Figure 11) [56]. By using the HPLC-DAD-MS^n^ coupling technique, 3 compounds of CR (ferulic acid, senkyunolide I, and senkyunolide H) and 8 kinds of compounds of GE (gastrodin, s-(4-hydroxybenzyl)-glutathion, parishin, parishin B, parishin C, p-hydroxybenzaldehyde, etc.) were identified with a fingerprint of Dachuanxiong Decoction [57]. Additionally, 17 different kinds of structure of Dachuanxiong Decoction (gastrodin, senkyunnolide I, senkyunnolide H, E-ligustilide, Z-Ligustilide, butylphthalide, riligustilide, tokinolide B, and levistolide A) were reported by the use of the LC-QTOF/MS technique in another research of Dachuanxiong Decoction [58]. Analyzing the statistics of Dachuanxiong Decoction *in vivo,* scientists demonstrated that there were 10 more different kinds of compounds, including 6 original substances of CR and 4 original substances of GR [59]. Additionally, gastrodin and parishin are definitely detected by serum pharmacochemistry in rat plasma after the gavage of Dachuanxiong Decoction, and those data help us establish a unified and advanced method to study DCXF [60].

Currently, some researchers have made some beneficial attempts to investigate the active ingredients behavior *in vivo* before and after the compatibility of the DCXF. Compared with the pharmacokinetic behaviors of rats *in vivo* before and after CR combined with GR, the area of GR in the brain tissue of rat drug concentration curve of CR-GR group was 1.66 times larger than GR only used group, and the mean residence time of was CR-GR group was longer and the clearance is 64.58% of GR only used group, which will significantly illustrate that DCXF could increase the bioavailability and slow down the elimination rate of GR [61]. Another recent study stated that the hydrolytic metabolites of ligustilide had been found in Dachuanxiong Decoction administration group, while there was no signal in the ligustilide administration group. It is suspected that the discovery of new related metabolite may be related to the interaction of the effective substance in the DCXF [50].

## 4. Pharmacological Effects

In recent years, the preparations based on DCXF have been used widely for angioneurotic headache and is notable on both pharmacological action and clinical effect resulting from blood stasis. Therefore, the pharmacological actions of DCXF have been further researched and discussed by scholars.

Migraine, a multivariate complex disease, have a high incidence and could be easily to recur. Dachuanxiong Decoction is always the drug of choice for treating migraines. According to different pathogenesis types, DCXF has been mainly studied for four aspects as follows.

### 4.1. Vascular Actions

At the onset of migraine, it has been demonstrated that some disturbances occur in the intra- and extracranial vascular contraction-dilation functions. Alkaloids could affect the contents of nitric oxide, nervous system (NOS), superoxidase dismutase, and methylene dioxyamphetamine of rats with focal cerebral ischemia [62]. Tetramethylpyrazine, an active ingredient of CR, had been assessed as it could protect endothelial cells injured by ox-LDL. The ratio of intimal or medial thickness and the number of monocytes in intimal could be significantly reduced by tetramethylpyrazine. Meanwhile, the MCP-1 and ICAM-1 levels in plasma and inhibited LOX-1 expression in the rabbit aortas were decreased after the treatment of tetramethylpyrazine. *In vitro* study revealed that monocyte adhesion to rat aortic endothelial cells was suppressed, rat aortic endothelial cell migration was inhibited, and MCP-1 and ICAM-1 expression in ox-LDL-injured RAECs were downregulated by treating with tetramethylpyrazine, which produced a certain degree of protection in atherosclerosis and endothelial cells [63]. For ischemic stroke, tetramethylpyrazine was also reported to have a global inhibitory effect on intracerebral cellular inflammatory response in a rat model of permanent cerebral ischemia [64]. Furthermore, the effects of tetramethylpyrazine on the fever could increase the plasma levels of tumor necrosis factor-*α* and the hypothalamic levels of glutamate, hydroxyl radicals, and prostaglandin-E2 induced by lipopolysaccharide. Reports also demonstrated that, by intravenous or intracerebroventricular tetramethylpyrazine 1 h after lipopolysaccharide injection, tetramethylpyrazine could attenuate lipopolysaccharide-induced fever [65]. Early research showed that DCXF had a better relaxing effect than a Chinese herbal monomer tetramethylpyraine. So it was obvious that the mutual promotion of DCXF was verified [66]. With the development of research, Dachuanxiong Decoction was demonstrated to be able to significantly decrease the vasodilatation of artery and vein vascular of dura mater in migraine model rats and to have obvious therapeutic actions of preventive effect of learning and memory impairment in the vascular dementia field [67, 68]. By using Dachuanxiong soft capsule, the whole blood, plasma viscosity, and fibrinogen of rats were reduced, prothrombin time was prolonged, and aggregation and adhesion of platelet were decreased [69]. Moreover, DCXF also had protective effects on the rats with focal cerebral ischemia and reperfusion compared with the control group intragastrically perfused with saline, and the probable mechanism was that DCXF could upgrade the expression of vascular epithelial growth factor [70–72].

### 4.2. Trigeminal Vascular Actions

Angiogenic substances could activate transmitters to stimulate the trigeminal nervous system (NOS), such as nitric oxide synthase and calcitonin gene-related peptide (CGRP) [68, 73]. It was demonstrated that phthalide compounds could obviously improve the behavior disorder of middle cerebral artery occlusion rats by reducing cerebral ischemia and inhibit platelet aggregation [74]. Ligustilide, an active components in CR, has anti-inflammatory activites on experimental ovariectomized osteopenic rats, and its anti-inflammatory potential was showed up in the regulation of nuclear factor kappa B, maleic dialdehyde, polymorphonuclear cells, interleukin-1*β*, inducible nitric oxide synthase and tumor necrosis factor-*α*, adhesion molecule, and cyclooxygenase-2 [75]. Also, tetramethylpyrazine exhibited a neuroprotective effect against ischemic deficits by reduction of behavioral disturbance, brain infarction, and edema, which might actively mediate neuroprotection against cerebral ischemia in both the endogenous defense capacity promotion and the attenuation of the extent and composition percentage of the major cellular inflammatory responses by targeting of macrophages or microglia by elevating Nrf2/HO-1 expression [64]. By using nitroglycerin (NO) model, Dachuanxiong Decoction was shown to decrease migraine symptoms, for it could release the pain neurotransmitters by regulating and controlling the trigeminal nervous system [76].

### 4.3. Cortical Spreading Depression Actions

The activations of receptors in dural vascular such as substance P (SP), NOS, and CGRP were significantly inhibited after having Dachuanxiong Decoction, such that the migraine was released [68]. It was reported that phenols in GR had antianxiety effects on mice [77]. Gastrodin, an effective constituent of phenols in GR, could significantly reduce CGRP-ir (+) neuron, CGRP-mRNA, and pERK1/2 expression level in rats and these actions were similar to the effective concentration of sumatriptan succinate [78]. Tetramethylpyrazine had been suggested could protect neural system by inhibit the expression of chemokine receptor 4(CXCR4). Thus, it is reasonable to create a new insights into therapeutic potential of tetramethylpyrazine in the treatment of migraine [79]. Ferulic acid, an active compound of phthalide in CR, was reported to have some effect on curing peripheral nerve injury for it could lead to a remarkable CGRP staining of the lamina I-II regions in the dorsal horn ipsilateral to the injury, recruit a significantly diminished number of macrophages, and shorten a significantly latency and an acceleration of the nerve conductive velocity of the evoked muscle action potentials [80]. Recently, it was proven that DCXF could reduce the abnormal increased content of CGRP and endothelin in the plasma of migraine patients during the attack stage and could also inhibit plasmatic extravasation, cerebrovascular abnormal expansion, and neurogenic inflammation caused by platelet activation [81]. Furthermore, the sedation and analgesia of DCXF was one of the mechanisms of relieving the headache and treating migraine [82]. 

### 4.4. Stimulating Cerebral Neuron Actions

In cerebral neuron theory, NO has participated in many cellular signaling transductions. Scientists investigated that tetramethylpyrazine had some effect on neuropathic pain-associated behaviors and neuronal apoptosis in the spinal dorsal horn and the results suggested that tetramethylpyrazine-induced analgesia inhibit, the neuronal apoptosis via the modulation of Bcl-2 and caspase-3 proteins in the rat spinal dorsal horn [83]. Also, tetramethylpyrazine exhibited a neuroprotective effect against ischemic deficits by reduction of behavioral disturbance, brain infarction, and edema, which might actively mediate neuroprotection against cerebral ischemia in both the endogenous defense capacity promotion [64]. Ligustilide had been reported to possess some neuroprotective effects on transient forebrain ischemia and permanent focal ischemia. After the investigation of the protective effects of ligustilide on parietal cortex and hippocampus of rats in chronic cerebral hypoperfusion mode, ligustilide showed obvious neuroprotective potential for treating chronic cerebral hypoperfusion injury, which might be attributed to its antiapoptosis of neuron and antiproliferation of astrocyte both in cortex and in hippocampus of the rats, suggesting that ligustilide might make some contribution to the prevention of migraine [84]. By evaluating the analgesic and antimigraine activities of senkyunolide I from CR, researchers suggests that senkyunolide I may be an active component of treating migraine. It was further explained that the mechanism of the relieve of pain in migraine model rats may caused by the adjustment of their turnover rates and the levels of monoamine neurotransmitters, as well as the decrease of nitric oxide (NO) levels in blood and brain [85]. By observing NO concentration in glial cells, Dachuanxiong Decoction is investigated to give rise to the variation of 5 hydroxytryptamine (5-HT) in glial cells by changing the concentration of NO in different doses. Additionally, it could increase and irritate 5-HT1D receptors in glial cells [86, 87], expand arterioles, and excite neurons [88]. By nourishing nerve cells and preventing calcium ions moving into nerve cells at the same time, DCXF could improve the viability and activities of nerve cells, decrease the intracellular free calcium concentration, and resist neuronal apoptosis [15, 89, 90]. 

### 4.5. Other Working Elements in Dachuanxiong Decoction

By exploring the dose-effect relationship of ferulic acid on ameliorating pain-depression dyad, scientists had demonstrated a clinical pain-depression dyad therapy of ferulic acid and suggested that ferulic acid can reverse pain-depression dyad, which could influence oxidative stress and monoamine level [91]. The present study was carried out to elucidate a centrally acting muscle relaxant effect of chloroform soluble fraction and its component, namely, ligustilide, cnidilide, and senkyunolide obtained from the rhizome of CR. As a curare-like action was not observed, a muscle relaxation induced by these phthalide compounds is considered to be due to central origin [92]. Thus, it is possible that ferulic acid might help release the pain of migraine by influencing oxidative stress and monoamine level, while ligustilide, cnidilide, and senkyunolide also contribute to the treatment of migraine by muscle relaxation.

Since ancient times, doctors of TCM have protected the dose as a secret part of treatment that should not be given to the public. Although the same herbal drug is used in different dose proportions, there are different efficacies with its dose combinations. From the historical evolution of drug use, scholars usually adjust the ratio of Chinese herb prescriptions on the basis of individual conditions. Consequently, the optimum proportion of DCXF has been investigated in recent years. By investigating its different proportions, the results confirmed that the ratio of CR-GR (CR : GR = 1 : 4) was the best compatibility [93]. After observing the effects of the cortex neuron cytoplasm of rats with different compatibility ratios, scientists demonstrated that the optimum proportion could notably lower the content of cytoplasm (Ca^2+^) of neuron in both normal oxygen supply and anoxic conditions [93]. Dachuanxiong Decoction is proved to have effects on acute blood-stasis and the influence for the blood velocity of rabbits common carotid in different ratios of CR and GR, which have no effects on the condition of only using GR [94]. 

Furthermore, researchers discovered that there are different effect pathways of CR and GR in DCXF. For CR, it occurred on vessel, while for GR it was on a nerve. Ultimately, it stimulated synergistic effects and enforced efficiency both by simultaneous use [94].

All these pharmacological effects are responsible for its significant therapeutic effects on migraine, vertigo, pains in rheumatoid arthritis, numb limbs, neck stiffness, attention deficit, and memory problems.

## 5. Prospects and Discussion

In summary, as per the review presented herein, DCXF, the abbreviation of Da Chuanxiong Formula, is an important traditional medicinal pair as well as formula in traditional Chinese medicinal systems for a long time. Organic acids, phthalides, phenols, polysaccharides, ceramides, cerebrosides, and microelements are believed to be the main bioactive constituents of DCXF. The herb pair exhibits significant clinical effects on antimigraine, neuron protection, and heart and cerebral functions improvement, with an abundant TCM connotation to balance the liver, extinguish wind, promote blood, and dispel wind. 

According to the basic theory of TCM prescription, the purpose of pair TCM herbs is to produce synergistic effects to enhance therapeutic efficacy and/or to minimize toxicity and adverse effects. For DCXF to increase pharmacological effects might be a main reason using CR and GR together. In spite of TCM theory illustration on its compatibility effects, in which CR and GR would be responsible for activating blood circulation and repressing *liver yang*, its compatibility mechanism is still rare to reveal right now. Despite the uncovery of increasing chemical compounds in CR and GR, some compounds such as ferulic acid and gastrodin are considered to be active constituents, for the reason of high contents in herbs and absorption compounds in blood [59]. However, these supposed active compounds could not represent DCXF. The herbal decoction generally has multiple components acting on multiple targets. So far, the real active compounds for each effective target have not been revealed, as well as the change of compounds contents in herbal decoction and pharmacokinetical behaviour in vivo after their compatibility. To investigate the interaction among compounds from CR and GR in terms of quality and quantity between single herb and formula would be the key to explain their synergistic effects.

In addition, in spite of hundreds of years application history safety studies of DCXF also should be scientifically performed, owing to high risk of cerebrovascular disease. It is of utmost urgency that more extensive clinical research, especially using randomized double-blind placebo-controlled crossover studies, should be carried out. Furthermore, more investigation is also needed to research on the effective components types, the differences and interaction in DCXF, the differences in terms of quality and quantity between CR, GR, and DCXF, the amount and the behavior of absorption, distribution, metabolism, excretion *in vivo.*

This review gives a brief summarization of recent research advances of DCXF, in the hope to provide some significant references for the study of DCXF, even for the compatibility of herb pair in TCM. Such an analysis will also help to identify the research gaps for generating novel drug delivery system. Thus, the data and studies presented in this review will be helpful for new product planning, R&D investment evaluation, and R&D productivity measurements.

## Figures and Tables

**Figure 1:**
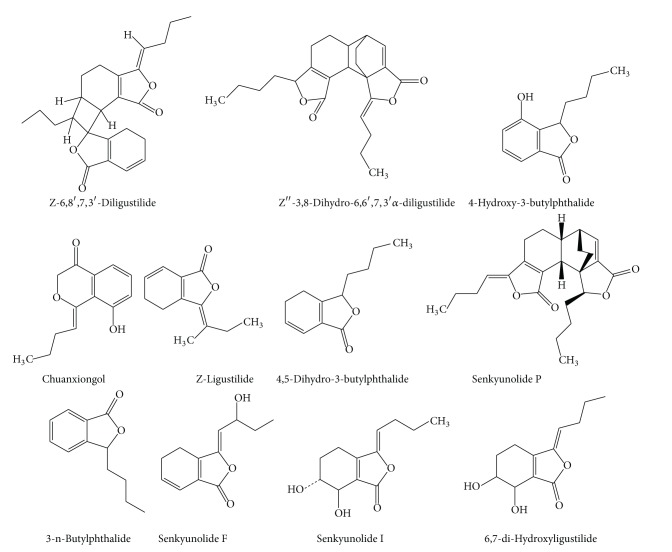
The chemical structures of phthalide compounds of CR.

**Figure 2:**
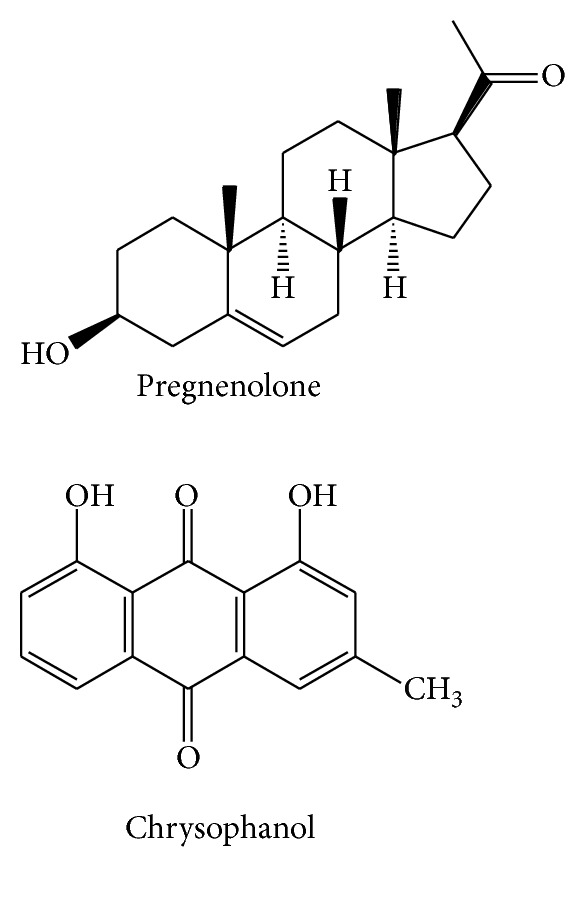
The chemical structures of organic acids of CR.

**Figure 3:**
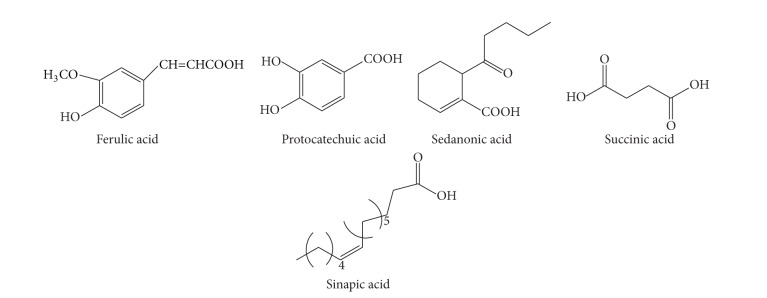
The chemical structures of phenols of CR.

**Figure 4:**
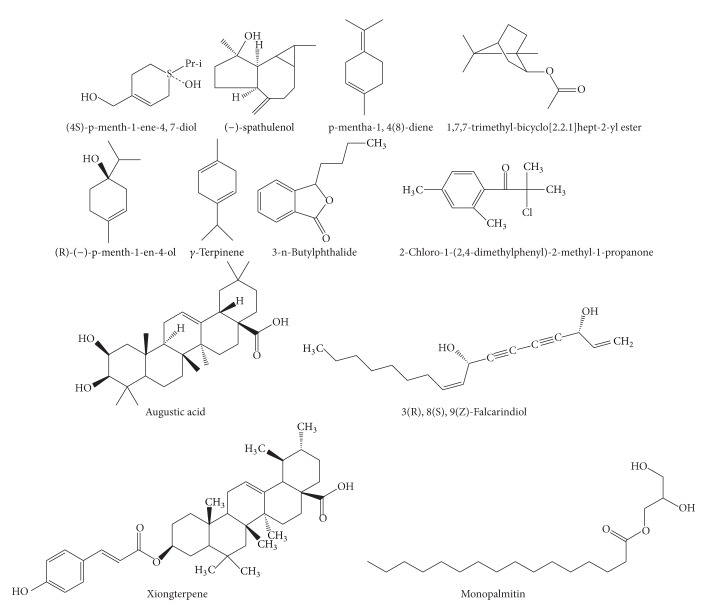
The chemical structures of essential oil compounds of CR.

**Figure 5:**
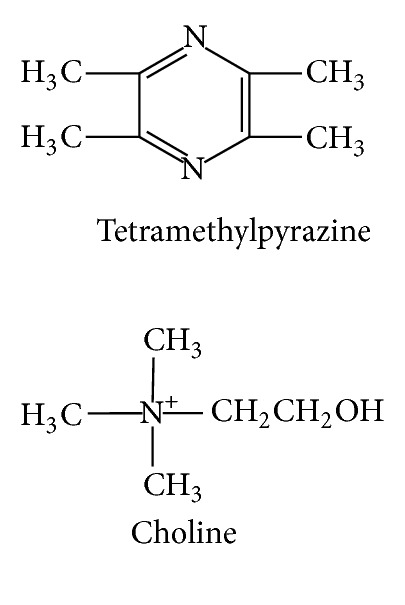
The chemical structures of alkaloid compounds of CR.

**Figure 6:**
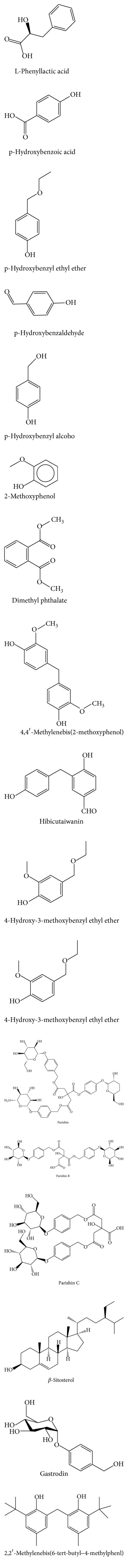
The chemical structures of phenols of GR.

**Figure 7:**
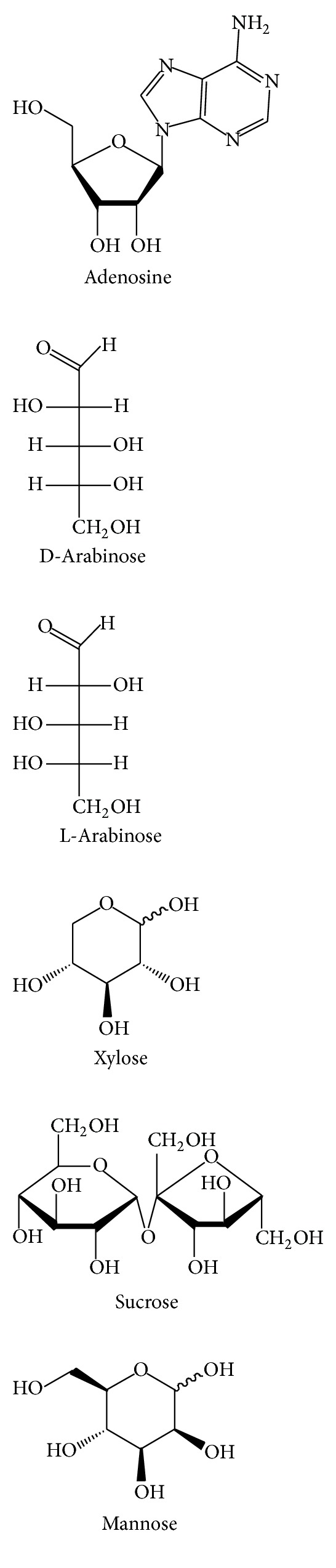
The chemical structures of polysaccharides of GR.

**Figure 8:**
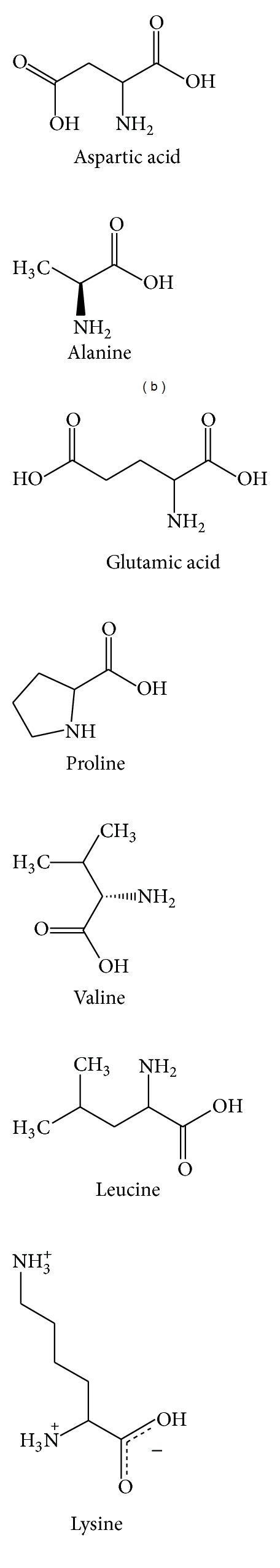
The chemical structures of microelement of GR.

**Figure 9:**
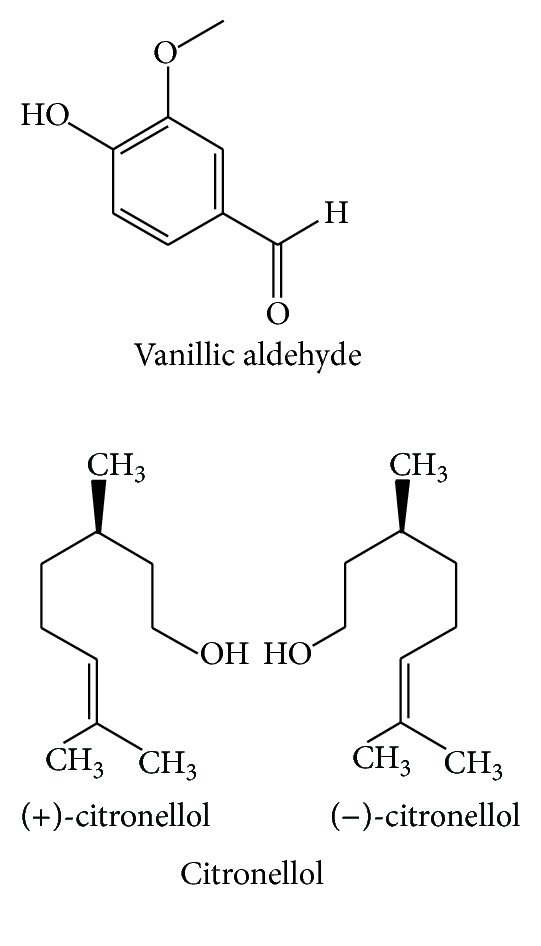
The chemical structures of organic acids of GR.

**Figure 10:**
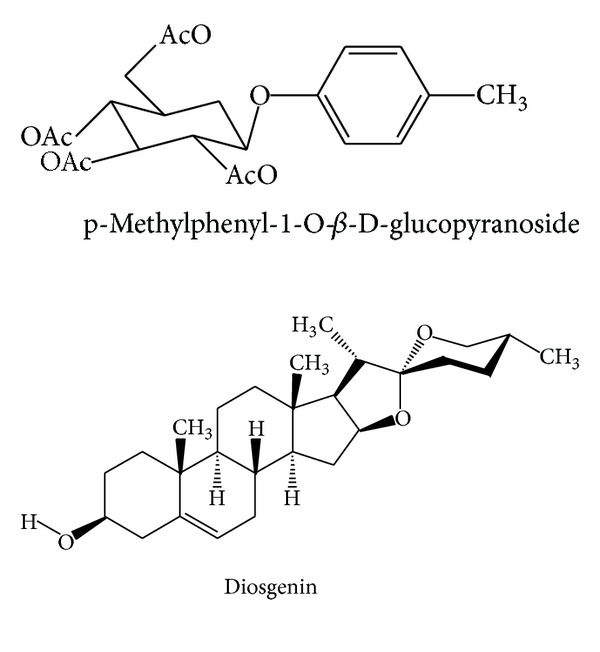
The chemical structures of other compounds of GR.

**Table 1:** Main chemical constituents and relative detection methods of CR.

Compound type	Compound name	Extract	Detection method	References
Phthalide compounds (Figure 1)	Z-6,8′,7,3′-Diligustilide	Ethanol	^ 1^H-NMR/^13^C-NMR	[16]
		Diethyl ether	^ 13^C-NMR	[17]
		Ethanol	^ 1^H-NMR/^13^C-NMR	[16]
		60% ethanol	LC-ESI-MS	[18]
		95% ethanol	TOF-MS/NMR	[19]
		Diethyl ether	^ 13^C-NMR	[17]
	Z′′-3,8-Dihydro-6,6′,7,3′*α*-diligustilide	Diethyl ether	^ 13^C-NMR	[17]
	4-Hydroxy-3-butylphthalide	80% methano	HPLC	[20]
		Acetic ether	MS	[21]
		95% ethanol	TOF-MS/NMR	[19]
	Ligustilide	Acetic ether	GC-MS	[22]
		80% methanol	HPLC	[20]
		60% ethanol	LC-ESI-MS	[18]
		Ethanol	^ 1^H-NMR/^13^C-NMR	[16]
	Senkyunolide F	Acetic ether	MS	[21]
	Senkyunolide I	95% ethanol	TOF-MS/NMR	[19]
		Acetic ether	MS	[21]
	Senkyunolide P	60% ethanol	LC-ESI-MS	[18]
	4,5-Dihydro-3-butylphthalide	Diethyl ether	^ 13^C-NMR	[17]
	Chuanxiongol	80% methanol	HPLC	[20]
	6,7-di-Hydroxyligustilide	80% methanol	HPLC	[20]
	3-n-Butylphthalide	60% ethanol	LC-ESI-MS	[18]
		Acetic ether	GC-MS	[22]

Organic acids (Figure 2)	Feulic acid	Ethanol	^ 1^H-NMR	[23]
		95% ethanol	NMR	[19]
		Ethanol	IR/^1^H-NMR	[24]
		80% methanol	HPLC	[20]
		60% ethanol	LC-ESI-MS	[18]
	Caffeic acid	95% ethanol	NMR	[19]
		Ethanol	IR/^1^H-NMR	[24]
		80% methanol	HPLC	[20]
	Succinic acid	95% ethanol	NMR	[19]
	Sadanic acid	Ethanol	IR/^1^H-NMR	[24]
	Sinapic acid	Ethanol	^ 1^H-NMR	[23]
	Protocatechuic acid	80% methanol	HPLC	[20]
	Augustic acid	Acetic ether	MS	[21]

Phenols (Figure 3)	Chrysophanol	Ethanol	IR/^1^H-NMR	[24]
	Pregnenlone	Ethanol	^ 1^H-NMR	[23]
		95% ethanol	NMR	[19]
		Acetic ether	MS	[21]
		95% ethanol	NMR	[19]
		Water	EI-MS/FAB-MS	[21]

Essential oil (Figure 4)	Xiongterpene	Acetic ether	MS	[21]
		95% ethanol	NMR	[19]
	(4S)-p-menth-1-ene-4,7-diol	Acetic ether	MS	[21]
		Water	EI-MS/FAB-MS	[21]
	3(R), 8(S), 9(Z)-Falcarindiol	Acetic ether	MS	[21]
		Water	EI-MS/FAB-MS	[21]
	1,7,7-Trimethyl-bicyclo[2.2.1]hept-2-yl	Acetic ether	GC-MS	[22]
	b-Phellandrene	Acetic ether	GC-MS	[22]
	*γ*-Terpinene	Acetic ether	GC-MS	[22]
	(R)-(−)-p-menth-1-en-4-ol	Acetic ether	GC-MS	[22]
	p-Mentha-1,4(8)-diene	Acetic ether	GC-MS	[22]
	Monopalmitin	95% ethanol	NMR	[19]
	(−)-Spathulenol	Acetic ether	GC-MS	[22]
	2-Chloro-1-(2,4-dimethylphenyl)-2-methyl-1-propanone	Acetic ether	GC-MS	[22]

Alkaloid (Figure 5)	Tetramethylpyrazine	80% methanol	HPLC	[20]
	Choline	80% methanol	HPLC	[20]

**Table 2:** Main chemical constituents and relative detection methods of GR.

Compound type	Compound name	Extract	Detection method	References
Phenols (Figure 6)	Gastrodin	Water	^ 1^H-NMR/^13^C-NMR	[35]
		Ethanol	HPLC	[36]
	p-Hydroxybenzyl alcohol	70% ethanol	EI-MS/^1^HNMR/^13^CNMR	[37]
		Water	^ 1^H-NMR/^13^C-NMR	[35]
	p-Hydroxybenzyl ethyl ether	70% ethanol	EI-MS/^1^HNMR/^13^CNMR	[37]
		Water	^ 1^H-NMR/^13^C-NMR	[35]
	p-Hydroxybenzoic acid	Water	^ 1^H-NMR/^13^C-NMR	[35]
	4-Hydroxy-3-methoxybenzyl ethyl ether	Water	^ 1^H-NMR/^13^C-NMR	[35]
	p-Hydroxy-benzyl-methyl ether	70% ethanol	EI-MS/^1^HNMR/^13^CNMR	[37]
		Water	^ 1^H-NMR/^13^C-NMR	[35]
	p-Hydroxybenzaldehyde	Water	^ 1^H-NMR/^13^C-NMR	[35]
		70% ethanol	EI-MS/^1^HNMR/^13^CNMR	[37]
	L-Phenyllactic acid	Water	^ 1^H-NMR/^13^C-NMR	[35]
	Hibicutaiwanin	Water	^ 1^H-NMR/^13^C-NMR	[35]
	4,4′-Methylenebis	Water	^ 1^H-NMR/^13^C-NMR	[35]
	2-Methoxyphenol	Water	^ 1^H-NMR/^13^C-NMR	[35]
	Parishin	Water	^ 1^H-NMR/^13^C-NMR	[35]
	Parishin B	Water	^ 1^H-NMR/^13^C-NMR	[35]
	Parishin C	Water	^ 1^H-NMR/^13^C-NMR	[35]
	*β*-Sitosterol	70% ethanol	EI-MS/^1^HNMR/^13^CNMR	[37]
	2,2′-Methylenebis (6-tert-butyl-4-methylphenl)	70% ethanol	EI-MS/^1^HNMR/^13^CNMR	[37]
	Dimethyll phthalate	70% ethanol	EI-MS/^1^HNMR/^13^CNMR	[37]

Polysaccharides (Figure 7)	Adenosine	70% ethanol	EI-MS/^1^HNMR/^13^CNMR	[37]
	Arabinose	70% ethanol	EI-MS/^1^HNMR/^13^CNMR	[37]
	Mannose	70% ethanol	EI-MS/^1^HNMR/^13^CNMR	[37]
	Xylose	70% ethanol	EI-MS/^1^HNMR/^13^CNMR	[37]
	Sucrose	70% Ethanol	EI-MS/^1^HNMR/^13^CNMR	[37]

Microelement (Figure 8)	Aspartic acid	Dry powder	Automatic Amino-acid Analyzer	[38]
	Alanine	Dry powder	Automatic Amino-acid Analyzer	[38]
	Glutamic acid	Dry powder	Automatic Amino-acid Analyzer	[38]
	Proline	Dry powder	Automatic Amino-acid Analyzer	[38]
	Valine	Dry powder	Automatic Amino-acid Analyzer	[38]
	Lysine	Dry powder	Automatic Amino-acid Analyzer	[38]
	Leucine	Dry powder	Automatic Amino-acid Analyzer	[38]

Organic acids (Figure 9)	Vanillic aldehyde	Ethanol	HPLC	[36]
	Citronellol	Ethanol	HPLC	[36]

Other (Figure 10)	p-Methylphenyl-1-O-*β*-D-glucopyranoside	Water	^ 1^H-NMR/^13^C-NMR	[35]
		70% ethanol	^ 1^H-NMR/^13^C-NMR	[37]
	Methyl-O-*β*-D-glucopyranoside	Water	^ 1^H-NMR/^13^C-NMR	[35]
	Diosgenin	Water	^ 1^H-NMR/^13^C-NMR	[35]
